# Evidence for adaptation of porcine Toll-like receptors

**DOI:** 10.1007/s00251-015-0892-8

**Published:** 2015-12-23

**Authors:** Kwame A. Darfour-Oduro, Hendrik-Jan Megens, Alfred Roca, Martien A. M. Groenen, Lawrence B. Schook

**Affiliations:** Department of Animal Sciences, University of Illinois, Urbana-Champaign, IL 61801 USA; Animal Breeding and Genomics Centre, Wageningen University, Droevendaalsesteeg 1, Wageningen, 6708 PB The Netherlands

**Keywords:** TLR, Porcine, Local adaptation, Immunity, Infectious diseases

## Abstract

Naturally endemic infectious diseases provide selective pressures for pig populations. Toll-like receptors (*TLRs*) represent the first line of immune defense against pathogens and are likely to play a crucial adaptive role for pig populations. This study was done to determine whether wild and domestic pig populations representing diverse global environments demonstrate local *TLR* adaptation. The genomic sequence encoding the ectodomain, responsible for interacting with pathogen ligands of bacterial (*TLR1*, *TLR2* and *TLR6*) and viral (*TLR3*, *TLR7* and *TLR8*) receptors, was obtained. Mitochondrial D-loop region sequences were obtained and a phylogenetic analysis using these sequences revealed a clear separation of animals into Asian (*n* = 27) and European (*n* = 40) clades. The *TLR* sequences were then analyzed for population-specific positive selection signatures within wild boars and domesticated pig populations derived from Asian and European clades. Using within-population and between-population tests for positive selection, a *TLR2*-derived variant 376A (126Thr), estimated to have arisen in 163,000 years ago with a frequency of 83.33 % within European wild boars, 98.00 % within domestic pig breeds of European origin, 40.00 % within Asian wild boars, and 11.36 % within Asian domestic pigs, was identified to be under positive selection in pigs of European origin. The variant is located within the N terminal domain of the *TLR2* protein 3D crystal structure and could affect ligand binding. This study suggests the *TLR2* gene contributing to responses to bacterial pathogens has been crucial in adaptation of pigs to pathogens.

**Electronic supplementary material:**

The online version of this article (doi:10.1007/s00251-015-0892-8) contains supplementary material, which is available to authorized users.

## Introduction

European and Asian wild boars diverged 1.6–0.8 million years (Myr) ago (Frantz et al. 2013) with domestication events occurring approximately 9000 years ago (Giuffra et al. 2000). European wild boars and European domesticated pigs and their Asian counterparts may have experienced different selective pressures given that they have originated from different continents with different endemic infectious diseases (Troy et al. 2001; Novembre and Di Rienzo 2009). Such differences in selective pressures can be exploited to detect immune-related genes that have been of adaptive value in terms of disease resistance within pig populations.

The vertebrate immune system is comprised of the innate and acquired immune components. The innate immune component is ancient and characterized by rapid and non-specific responses to pathogenic microbes while the acquired immune component is characterized by highly specific memory responses. The Toll-like receptor (*TLR*) family represents a class of innate immunity receptors important during early phases of infections that also serve as a link between the innate and acquired immune responses (Werling and Jungi 2003), consequently triggering inflammatory responses to prevent bacterial and viral spread. In vertebrates, 10–12 *TLRs* have been identified (Leulier and Lemaitre 2008) and are classified based on their cellular location and the type of pathogenic ligands they recognize. Cell surface expressed *TLRs* (*TLR1*, *TLR2*, *TLR4*, *TLR5*, and *TLR6*) recognize predominantly bacterial ligands and several fungal and parasite ligands while *TLR3*, *TLR7*, *TLR8*, and *TLR9* are expressed within endosomes and recognize single- and double-stranded RNA and CpG DNA (Akira et al. 2006). Single nucleotide polymorphisms (SNPs) and differences in expression of *TLR* molecules affect immune responses to numerous pathogens and are associated with host resistance to infectious diseases (Lazarus et al. 2002). Several non-synonymous substitutions have been detected in porcine *TLR* genes, especially in the ectodomain of cell surface-expressed *TLRs* (Uenishi et al. 2011), suggesting functional relevance of the ectodomain and specific SNPs residing within these regions.

Naturally occurring porcine diseases endemic to particular geographic locations include African swine fever prevalent in sub-Saharan Africa; the ancient foot and mouth disease endemic in some regions of Asia, most of Africa, and the Middle East; brucellosis observed in the Middle East, the Mediterranean region, sub-Saharan Africa, China, India, Peru, and Mexico; and swine influenza which is endemic to North and South America, Europe, and parts of Asia (OIE 2012). The occurrence of such locally or regionally endemic diseases suggests that long-term adaptation may have occurred between the host and pathogen (Zimmerman et al. 2012). Thus, such adaptation could be detected as genomic signatures across populations. Recent studies have shown that approximately 7 % of the pig genome has been influenced by selection either in the form of positive or balancing selection (Amaral et al. 2011). These signals were observed in genomic regions involved in coat color, brain and muscle development, growth, metabolism, olfaction, and immunity and were specific to certain pig populations (Amaral et al. 2011). Differences in amino acid substitutions and heterozygosity of *TLR* SNPs in European and Asian pigs have been observed and might be due to differences in pathogens encountered in the domestication and initial breed development environments of the pigs (Shinkai et al. 2006). Porcine *TLRs* therefore represent candidate immune genes for investigating pathogen-driven selective pressures specific to geographically defined populations.

The aim of this study was to determine whether adaptation to local environments of wild and domestic European and Asian pig populations resulted in *TLR* gene genomic signatures of selection. We hypothesized that geographically restricted selective pressures resulted in adaptive genomic signatures within porcine *TLRs*. To test this hypothesis, the sequences encoding the ectodomain of bacterial recognizing (*TLR1*, *TLR2*, and *TLR6*) and viral sensing (*TLR3*, *TLR7*, and *TLR8*) receptors were obtained and the following specific questions were addressed: (1) whether adaptive selection pressures on pigs from both European and Asian environments was evident and (2) whether different selective pressures for each geographic environment were identified.

## Materials and methods

### Study populations

Sixty-seven individuals consisting of wild boars and domestic pigs representing populations with origins from diverse geographic environments within Asia and Europe were utilized in this study. European wild boars were represented by 11 unrelated individuals from the Netherlands, 2 from Greece, and 1 each from Switzerland and Italy. European domestic breeds were sampled from unrelated pigs including Angler Sattleschwein (1), Mangalitsa (1), Durocs (4), Hampshires (3), Landraces (6), Pietrains (4), Charto Murciano (2), Cassertana (2), Retinto (1), and Cinta Senese (1). Asian wild boars were represented by individuals from Northern China (3), from Southern China (1), and from Japan (1). Finally, Asian domestic pigs included Meishan pigs (10), Jiangquahai pigs (3), Xiang pigs (2), Wannan spotted pigs (2), Jinhua pigs (2), Leping spotted pigs (2), and Zang pig (1). Animals within a group were not related (no shared grandparents). In addition, one species individual from the family Suidae (*Phacochoerus africanus* (Common warthog), *Sus verrucosus* (Javan warty pig), or *Sus celebensis* (Sulawesi warty pig) were also analyzed. The source of DNA samples have been previously described (Bosse et al. 2012). Supplementary Fig. S1 shows the geographic sampling regions.

### Loci analyzed

Six *TLR* genes chosen for this study are described in Supplementary Table S1. The TLRs ectodomains were examined since they are the functional sites involved in pathogen ligand recognition. The D-loop region of mtDNA, a neutral marker, was used to demonstrate the independent domestication origins of European and Asian pigs (Larson et al. 2005). The D-loop region of the mtDNA of each animal was obtained by direct sequencing and analyzed to obtain clustering patterns among the sampled animals. On each of the 18 autosomal chromosomes of pigs, one noncoding genomic region (2 kb in size) (Supplementary Table S2) was chosen to represent neutrally evolving regions. These noncoding genomic regions were at least 50 kb from any known or predicted porcine gene, as determined by inspecting the *Sus scrofa* 10.2 genome assembly on the UCSC genome browser. The 50-kb buffer was chosen to minimize the effect of linkage disequilibrium with genic SNPs (Wagh et al. 2012).

### DNA sequencing

DNA extraction, library preparation, and sequencing have been described previously (Bosse et al. 2012). Briefly, DNA was extracted from whole blood by using the QIAamp DNA blood spin kit (Qiagen Sciences), and quantity and quality parameters were performed on the Qubit 2.0 fluorometer (Invitrogen) and run on a 1 % agarose gel. Library construction and re-sequencing of individual pig samples utilized 1–3 μg of genomic DNA following Illumina library preparation protocols. The library insert size was 300–500 bp and sequencing was performed using the 100 paired-end sequencing kit. All DNA were sequenced to approximately 8× depth. Quality trimmed reads (phred quality >20, minimum length of pairs of reads = 40 bp) were aligned to the *S. scrofa* reference genome build 10.2 using the unique alignment option of Mosaik Aligner (V.1.1.0017). The aligned reads from each of the animals together with the *S. scrofa* reference genome were stored as bam files for each individual animal.

The coordinates of genes within the bam files were obtained by querying the *S. scrofa* reference genome build 10.2 with coding sequences of porcine TLRs from public databases. The accession numbers of sequences obtained from the public databases for TLRs were *TLR1*: NM_001031775, *TLR2*: NM_213761, *TLR3*: HQ412796, *TLR6*: NM_213760, *TLR7*: NM_001097434, and *TLR8*: ENSSSCG00000012118. Perl scripts were used to extract the TLR coding sequences and the noncoding genomic regions. Sequences were aligned using ClustalW 1.81 (Thompson et al. 1997). The D-loop region of mtDNA was obtained by direct sequencing.

A 715-bp fragment of the D-loop region was amplified by polymerase chain reaction (PCR). The nucleotide sequences of the primers used were as follows: forward 5′CTCCGCCATCAGCACCCAAAG3′ and reverse 5′GCACCTTGTTTGGATTRTCG3′ (Luetkemeier et al. 2010). Final reactions were made to a final volume of 12 μL containing each reaction PCR master mix (Thermo Fisher Scientific, USA) which supplies 1.5 mM MgCl_2_, 0.625 U of Taq DNA polymerase, and 0.2 mM dNTPs. Then, 5 pM of each primer and ~40 ng DNA were added to the final reaction. Amplification protocol was performed as follows: 35 cycles, each consisting of 95 °C for 30 s, 55 °C for 45 s, and 72 °C for 90 s and then a final extension at 72 °C for 10 min. The amplicon was confirmed using 2 μL PCR product by electrophoresis on a 1.5 % agarose gel stained with ethidium bromate visualized under UV light. The remaining PCR products were purified using a Multiscreen PCR 96 cleanup vacuum system (Millipore). Bidirectional sequencing reactions were carried out using Big-Dye Terminator Cycle Sequencing Ready Reaction kit v3.1 (Applied Biosystem, USA) in an ABI3730 DNA Analyzer sequencer (Applied Biosystem, USA). In order to correct sequencing errors and obtain the consensus sequence for each individual amplicon, GAP 4 was utilized (Bonfield et al. 1995), using the mtDNA pig sequence GenBank AJ002189 (Ursing and Arnason 1998) as a reference sequence.

### Data analysis

Porcine TLR reference amino acid sequences were aligned to the corresponding human sequences in order to delineate the ectodomain, the leucine-rich repeat (LRR) modules, ligand-binding and dimerization domains, and other sub-domains within the ectodomain (Supplementary Fig. S2). Haplotype reconstructions from the aligned sequences for all loci were carried out with PHASE 2.1.1 software (Stephens and Scheet 2005) using the SNiPLAY web-based tool for SNP and polymorphism analysis (Dereeper et al. 2011). For the D-loop region sequences of mtDNA, the Neighbor-Joining method (using p-distance) implemented in MEGA version 5 (Tamura et al. 2011) was used for phylogenetic analysis (Supplementary Fig. S3). A bootstrap of 1000 replicates was conducted. To test for departure from the standard neutral model of evolution, Tajima’s D, Fu and Li’s D*, and Fu and Li’s F* were conducted using DnaSP (Librado and Rozas 2009).

Derived allele under positive selection was determined using the derived intrallelic nucleotide diversity (DIND) test. The DIND test was applied by plotting for all SNPs within the ectodomain of genes within groups that showed deviation from neutrality, the ratio between the ancestral and derived internal nucleotide diversity (diversity among haplotypes carrying alleles) against the frequencies of derived alleles. An elevated ratio associated with a high derived allele frequency was used as an indication of positive selection of the derived allelic state. To define statistical significance, the values estimated for *TLRs* were then compared against the background neutral distribution obtained by means of 10,000 simulations of the sequences encoding the ectodomain conditional on the number of segregating sites and the recombination rate of the sequences and integrating a simplified version of a wild boar demographic model (initial effective population sizes of European and Asian wild boars = ~25,000, a bottleneck at 20,000 years ago, and an effective population size 10,000 years ago of 3000 for European wild boars and 13,500 for Asian wild boars) (Groenen et al. 2012). Simulations were carried out using Fastsimcoal2 (Excoffier et al. 2013). *TLR* SNPs that fell beyond 90th and 95th percentiles of the neutral distribution were considered to be under positive selection. Singletons were excluded from this analysis.

Interpopulation differentiation (F_ST_) and expected loci heterozygosity were calculated for *TLR* SNPs that were polymorphic in each of two populations being compared and intergenic region sequences SNPs using Arlequin ver 3.5 (Excoffier and Lischer 2010). *TLR* SNPs showing high levels of population differentiation and, thus, the target of positive selection were identified by comparison of *TLR* SNP F_ST_ values and the 90th and 95th percentiles for F_ST_ distribution (estimated using heterozygosity sliding windows of size 0.025 with increasing step of 0.01) computed for SNPs of the noncoding genomic regions. The *p* value for a SNP was estimated (Barreiro et al. 2009) where, first, F_ST_ values for a *TLR* SNP was compared with F_ST_ values from the noncoding genomic region sequences SNPs with an expected heterozygosity value of ±0.025 with respect to that observed for the *TLR* SNP. Then among the noncoding genomic region SNPs, the proportion of SNPs with F_ST_ values higher than that observed for the *TLR* SNP was used as the *p* value. Ancestral and derived states of *TLR* alleles were determined by a strategy (Groenen et al. 2012) where an allele is assumed to be ancestral when one of the alleles in *S. scrofa* was observed in *P. africanus* (Common warthog), *S. verrucosus* (Javan warty pig), or *S. celebensis* (Sulawesi warty pig) in that order, respectively. A maximum likelihood approach implemented in GENETREE version 9 (Griffiths and Tavare 1994) was used to estimate theta (ϴ = 4N_e_μ) and age of mutations. The default mutation rate (μ = 2.5 × 10^−8^) of humans was used as there is no known mutation rates for pigs (Groenen et al. 2012). Time estimated in generations (*T*) were converted into years (*t*) using a 5-year generation time (*g*) with the formula *t* = 2 × *N*_e_ × *T* × *g* as stated in the GENETREE manual. Median-joining phylogenetic haplotype networks were constructed based on SNPs within the TLRs using Network 4.6.1.1 (www.fluxus-technology.com). Only haplotypes present in a minimum of two animals were considered. MuPIT Interactive (Niknafs et al. 2013) was used to map variants under selection on to three-dimensional (3D) protein structures. Swiss prot and Ensemble genome browsers were utilized to determine the functional consequences of TLR sites under positive selection.

## Results

Sequences (67 sequences for each TLR alignment) encoding the ectodomains of bacterial sensing *TLR1*, *TLR2*, and *TLR6* and viral sensing *TLR3*, *TLR7*, and *TLR8* from wild boars and domestic pigs of European and Asian origins were obtained. The length of the sequences in terms of number of nucleotides of the TLRs ranged from 1668 bases for *TLR1* to 2445 bases for *TLR7*. Amino acid length ranged from 556 amino acids for *TLR1* to 792 amino acids for *TLR7*. A total of 136 SNPs were identified within the TLR sequences encoding the ectodomain (Supplementary Table S3). Majority of the SNPs have been previously reported (Shinkai et al. 2006; Morozumi and Uenishi 2009; Bergman et al. 2010).

### Evidence of positive selection pressure within population

To determine whether there is evidence of positive selective pressure mediated by infectious agents of endemic diseases on pig populations, wild boars and domestic pigs from previously defined European and Asian lineages were chosen (Megens et al. 2008). The geographic origins of these populations were validated by sequencing the mitochondrial D-loop regions of these animals and constructing a phylogenetic tree. The Neighbor Joining tree (Supplementary Fig. S3) obtained from analysis of the D-loop region sequences revealed two clades of animals representing animals of European and Asian origins. For analysis, animals were grouped by geographic origins and domestication status. The groups considered were therefore all European animals (wild boars and domestic pigs combined, *N* = 40), all Asian animals (wild boars and domestic pigs combined, *N* = 27), Asian wild boars (*N* = 5), European wild boars (*N* = 15), Asian domestic pigs (*N* = 22), and European domestic pigs (*N* = 25). Gene sequences of bacterial sensing *TLR1*, *TLR2*, and *TLR6* and viral sensing *TLR3*, *TLR7*, and *TLR8* for each animal were extracted from whole genome resequenced data from each animal. Analysis of positive selection focused on the ectodomains (Supplemetary Table S1) involved in pathogen recognition.

**Fig. 1:**
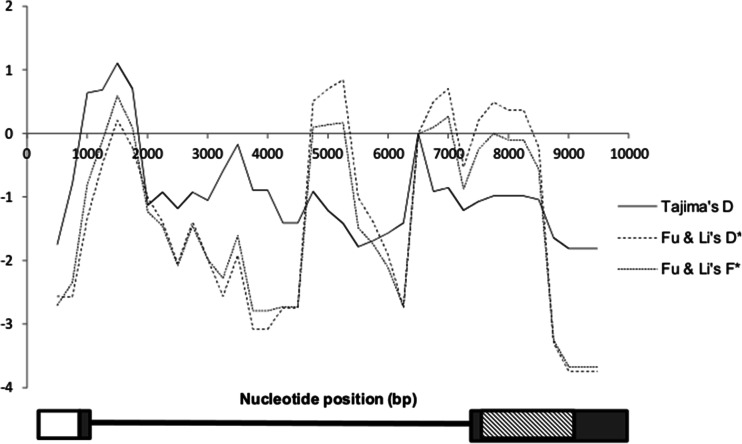
Sliding window plots for *TLR2* gene. Sliding window plots of Tajima’s D, Fu and Li’s F*, and Fu and Li’s D* tests within the European (wild boars and domestic pigs combined) porcine population using window length of 1000 bp and step size of 250 bp. The *white box* represents the five upstream regions, the *thick black line* represent the intronic region, the *grey boxes* show the exonic regions and the ectodomain is represented by the *box with light down diagonal lines*. The distal part of the ectodomain show significant values for neutrality indices

**Fig. 2:**
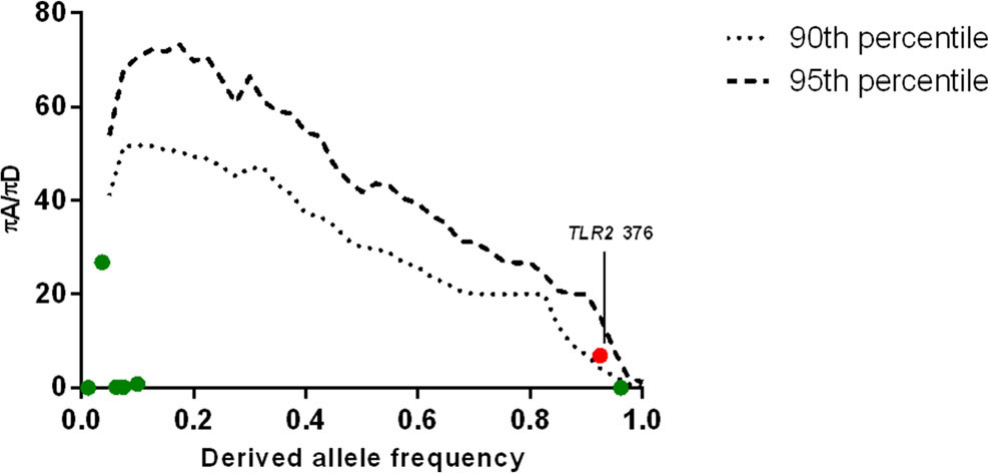
Positive selection for *TLR2*. Detection of positive selection in European (wild boars and domestic pigs combined) porcine population *TLR2* ectodomain using the DIND test. The *short and long dashed lines* represent the 90th and 95th percentiles of the empirical distribution of expected πA/πD values obtained from 10,000 simulations of the ectodomain conditional on the number of segregating sites and the recombination rate of the ectodomain and integrating the wild boar demographic model previously described (Groenen et al. 2012). *Green dots* represent *TLR2* SNPs under neutrality and *red dot* represents *TLR2* SNPs under positive selection

**Fig. 3:**
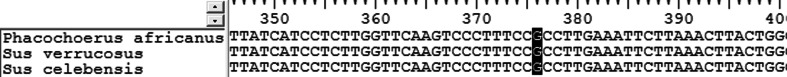
Alignment of *TLR2* sequences of Suidae species. Illustrated is the G allele at nucleotide position 376 within *TLR2* as the ancestral allele

The following tests were performed to determine evidence of adaptive selection pressure for European and Asian pig populations: (1) test of deviation from neutrality (sliding window analysis of Tajima’s D, Fu and Li’s D*, and Fu and Li’s F*) due to shift to a low frequency spectrum polymorphism; and (2) test for derived alleles under recent positive selection due to high frequency of the allele in a population (Rubin et al. 2012) using the DIND test (Barreiro et al. 2009). Given the relatively small population sample sizes, genes under selection were defined conservatively as those for which both neutrality and DIND test were significant in the same population (Manry et al. 2011). Using this stringent criteria, *TLRs* (*TLR1*, *TLR3*, *TLR6*, *TLR7*, and *TLR8*) investigated in this study did not show signatures of adaptive selection (data not shown) in any population. The stringent criteria employed here and the limited number of genes used in this study indicated that fewer number of SNPs were expected to show signatures of adaptive selection. Each of the three neutrality tests detected significant (*p* < 0.05) excess of rare alleles within the bacterial sensing *TLR2* exon 2 (encoding the ectodomain) of the European (wild boars and domestic pigs combined) population (Tajima’s D = −1.80; Fu and Li’s D* = −3.74; Fu and Li’s F* = −3.67, Fig. 1) consistent with positive selection or population expansion. Based on the DIND test involving SNPs within the *TLR2* sequences of the European population, the derived allele *TLR2* SNP 376A (126Thr), located on exon 2 of the *TLR2* gene with a frequency of 92.5 % (83.33 % within European wild boars and 98.00 % within domestic pig breeds of Europe), was detected as showing evidence of positive selection (πA/πD = 6.88; *p* = 0.055, Fig. 2). Three European wild boars were heterozygous, 1 wild boar was homozygous for the ancestral allele, whereas the 11 remaining wild boars were homozygous for the derived allele. One European landrace pig was heterozygous whereas all remaining European domestic pigs (*N* = 24) were homozygous for the derived allele. The derived allele is at a frequency of 40.00 % in Asian wild boars and 11.36 % in Asian domestic pigs population. Of the five Asian wild boars used in this study, one northern Chinese wild boar was homozygous for the derived allele, one northern Chinese wild boar and one Japanese wild boar were homozygous for the ancestral allele, whereas one southern Chinese wild boar and one northern Chinese wild boar were heterozygous. Seventeen Asian domestic pigs were homozygous for the ancestral allele. The remaining five Asian domestic pig breeds were heterozygous. Details on frequencies for TLR SNPs are as shown in Supplementary Table S3. Determination of the ancestral or derived state of an allele is described in the “Materials and methods” section. Figure 3 depicts a conservation of the G allele at *TLR2* position 376 within three wild pig relatives. According to the strategy employed by Groenen et al. (2012), the G allele is the ancestral allele and the A allele is derived. The change from the ancestral to the derived allele at *TLR2* SNP 376 is a nonsynonymous change (*TLR2* SNP G376A, Ala126Thr) and is likely to affect protein function.

### Selective pressure differences between populations

**Fig. 4:**
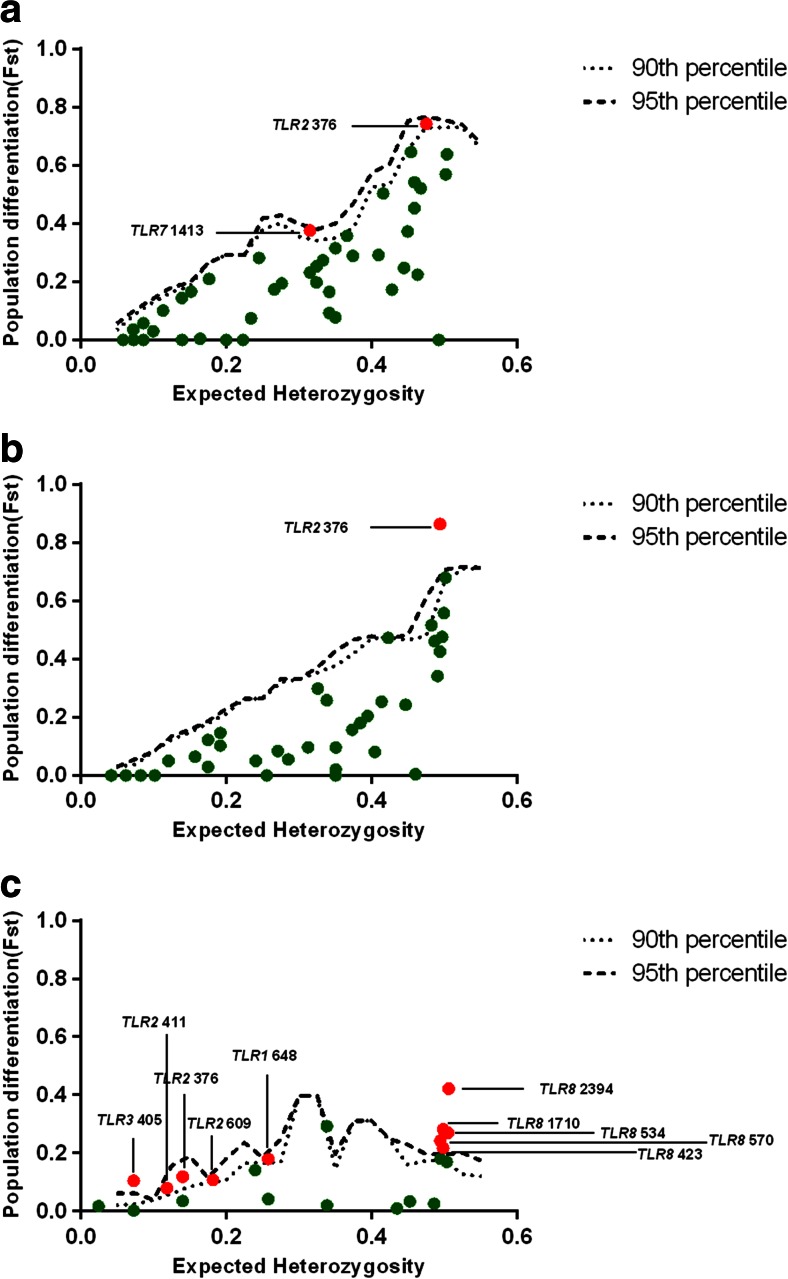
Positive selection detection at *TLR* SNPs on the basis of population differentiation. F_ST_ is plotted as a function of expected heterozygosity for every SNP between **a** Asian (wild boars and domestic pigs combined) versus European (wild boars and domestic pigs combined) porcine populations; **b** Asian domestic pig versus European domestic pig populations; and **c** European wild boar versus European domestic pig populations. *Dots* represent *TLR* SNPs. The *short and long dashed lines* represent the 90th and 95th percentiles of the empirical SNP distribution of F_ST_ of The noncoding genomic region sequences

To determine whether selective pressures were heterogeneous between populations, F_ST_ values for SNPs within TLR sequences were compared to the empirical distribution of F_ST_ from SNPs obtained from noncoding genomic region sequences chosen to represent neutrally evolving regions (see “Materials and methods”). The rationale behind this approach was that differences in selective pressure between populations could lead to elevated levels of population differentiation at immune genes relative to neutrally evolving loci (Barreiro et al. 2009). The nonsynonymous variant *TLR2* SNP G376A showed the highest level of population differentiation (F_ST_ between European domestic pigs and Asian domestic pigs = 0.86, *p* = 0.02 (Fig. 4a); F_ST_ between European pigs (wild boars and domestic pigs combined) and Asian pigs (wild boars and domestic pigs combined) = 0.74, *p* = 0.08 (Fig. 4b)). A comparison of European wild boars and European domestic pigs indicated that *TLR8* SNPs were highly differentiated relative to other *TLR* SNPs (Fig. 4c). However, the location of TLR8 gene on the X chromosome means it is prone to higher genetic drift which may result in elevated levels of population differentiation for *TLR8* SNPs (Barreiro et al. 2009). F_ST_ values between European wild boars and European domestic pigs were low relative to those between pigs of European and Asian origins, indicating weak differentiation between pig populations from the same geographic origin. Asian wild boar population was not compared to any other population in terms of F_ST_ given the small number of Asian wild boars involved in this study.

**Fig. 5:**
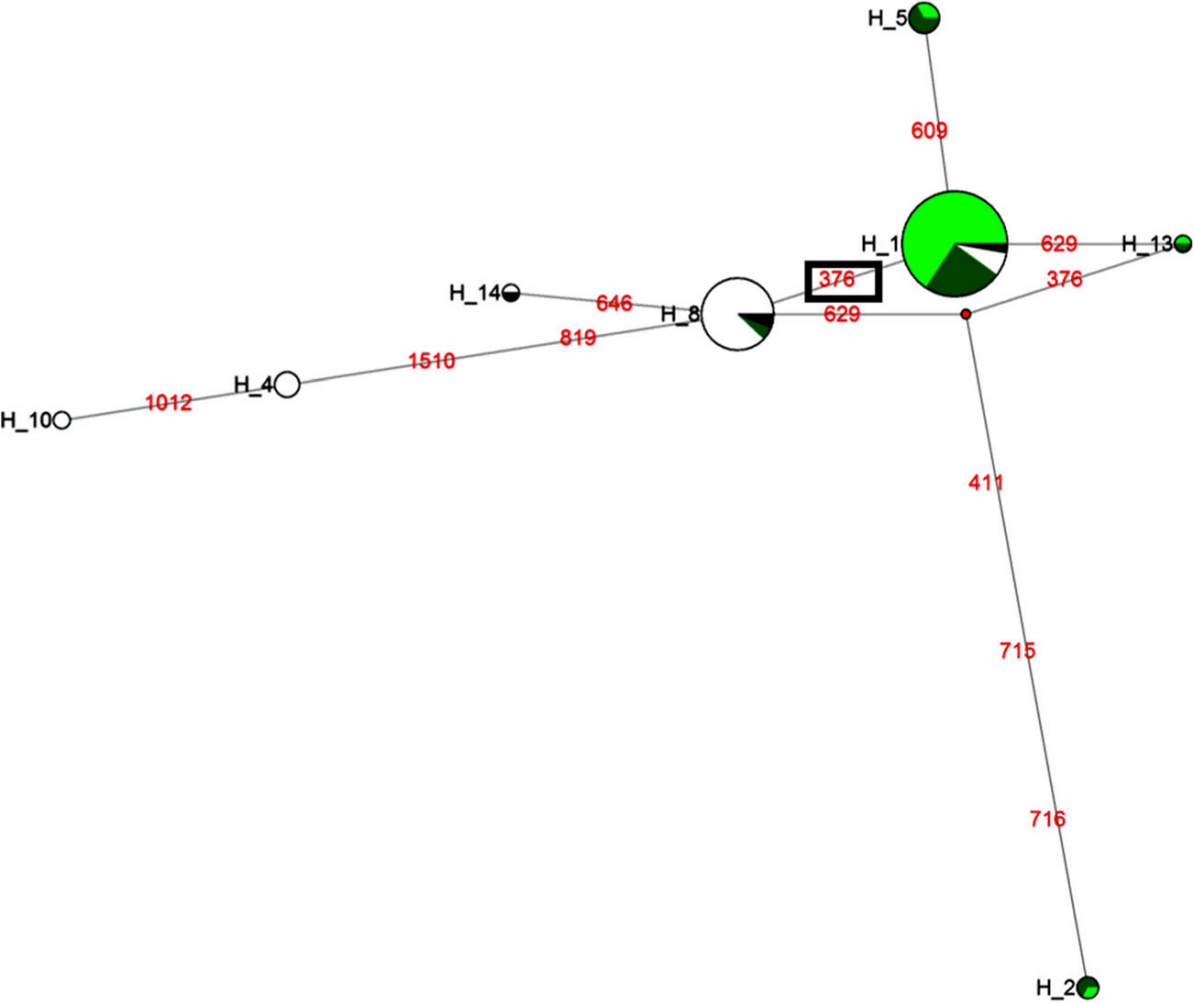
Median-joining network for haplotypes at the *TLR2* ectodomain. The *circles* represent haplotypes and the *size of the circles* are proportional to the frequency of the haplotypes. The mutation positions are shown along branches as *red numbers*. Asian wild boars, Asian domestic pigs, European wild boars, and European domestic pigs populations are shown in *black*, *white*, *deep green*, and *light green* colors, respectively. SNP 376, under positive selection is *boxed* and differentiates the Asian and European populations

To determine whether positive selection drives the prevalence of certain *TLR* haplotypes in European and Asian pig populations, haplotype reconstruction involving SNPs within the each *TLR* was done using the PHASE 2.1.1 software (Stephens and Scheet 2005) and the evolutionary relationships between haplotypes for each *TLR* were determined using a median-joining network. For *TLR2*, two high frequency haplotypes were observed and differed only at *TLR2* SNP 376 (Fig. 5, Supplementary Table S4), where the high frequency haplotype (H_1) dominated by the European individuals carried the derived SNP 376A allele and the high frequency haplotype (H_8) dominated by Asian individuals carried the ancestral SNP G376 allele. The high frequency haplotype within the European population was detected in all the European domestic pigs and the majority (10/15) of European wild boars. In order to estimate the divergence time of *TLR2* sequences encoding the ectodomain and the age of *TLR2* SNP G376A (Ala126Thr), maximum coalescence analysis by GENETREE (Griffiths and Tavare 1994) was utilized. Using all populations, the estimated time to most common ancestor (T_MRCA_) for the entire *TLR2* sequences geneology was 0.900 ± 0.28 Mya, which is close to the ~1 Mya since the split of the Asian and European wild boar, and the age of the 126Thr variant assuming neutrality was estimated to be 0.163 ± 0.08 Mya. The age of the derived allele and the presence of the haplotype carrying the derived allele in most European wild boars and all European domestic pigs involved in this study indicate that the allele arose within the wild boars, prior to the domestication process.

### Functional relevance of SNPs under positive selection

**Fig. 6:**
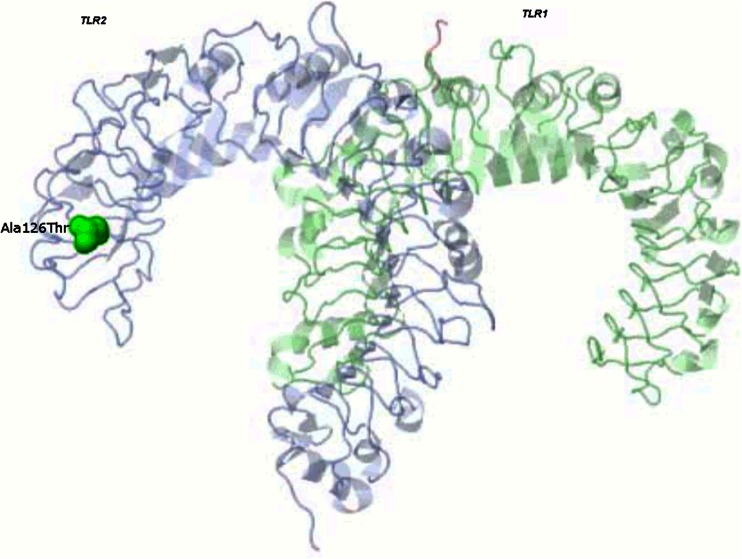
Location of amino acid residues under selection in *TLR2* 3D protein structure. Amino acid residue *TLR2* 126Thr under positive selection within the European (wild boars and domestic pigs combined) porcine population is shown within the crystal structure of *TLR1/TLR2* heterodimer (PDB ID 2Z7X) induced by binding of a tri-acylated lipopeptide

To determine whether variants under selection are within functional domains of TLR receptors, variants were mapped onto *TLR* protein 3D structure. The *TLR2* variant 126Thr is located within the N-terminal (on the fourth leucine-rich repeat (Supplementary Fig. S2)) and alpha helices of the *TLR2* protein and is surface exposed (Fig. 6), indicating a likely role in protein-protein interactions. Porcine *TLR2* amino acid sequence was also compared to human *TLR2* amino acid sequence from Swiss-Prot to examine whether the site under positive selection was within functionally relevant domains of the protein. *TLR2* variant 126Thr did not fall within any known region of functional region. Lastly, the *TLR2* variation table in Ensembl was examined to determine the effects of substitutions at *TLR2* amino acid site 126 on protein function. SIFT predicts whether an amino acid substitution affects protein function based on sequence homology and the physico-chemical similarity between alternate amino acids (Kumar et al. 2009). Substitutions with scores <0.05 are called “deleterious” and those with scores >0.05 are called “tolerated.” Within Ensembl, the substitution from Threonine to Alanine at porcine *TLR2* 126 (dbSNP identifier rs81218810) is predicted by SIFT to be deleterious (score = 0.03), indicating that substitution at the *TLR2* amino acid site 126 affects protein function.

## Discussion

This study supports the hypothesis that geographically restricted selective pressures on European and Asian wild boars that have diverged over 1 Mya have resulted in genomic signatures of adaptation in porcine *TLRs*. Specifically, *TLR2* SNP 376A (126Thr) of European pig (wild boars and domestic pigs combined) and not the Asian pig population showed evidence of positive selection, consistent with previous studies that have demonstrated that certain polymorphisms in *TLR2* sequences encoding the ectodomain of primates (Takaki et al. 2012), wild rodents (Tschirren et al. 2013), and cattle (Jann et al. 2008) involved in pathogen recognition have evolved adaptively conferring selective advantage. Porcine *TLR2* is found at the distal end of the q arm of Chromosome 8, in a region with identified QTLs for some immune related traits (Jann et al. 2009), and is therefore a potential target for positive selection. Furthermore, the association of *TLR2* with a wider panel of ligands and the need for heterodimerization (*TLR2* forms heterodimers with *TLR1* and *TLR6* to recognize lipopeptide components of gram-positive and gram-negative bacterial cell walls) indicates that the *TLR2* region experiences contrasting evolutionary actions, including adaptive evolution to the environment and pathogens (Jann et al. 2008). A comparison of the genomic coordinates [8:79,824,541-79,834,592] of porcine *TLR2* to recombination maps computed for four different pedigrees (Tortereau et al. 2012) indicate that Porcine *TLR2* lies in a region with a relatively low recombination rate on Chromosome 8. Regions with low recombination rate have been shown to be prone to positive selection (Barton 2000).

Within-population-based tests for positive selection utilized here have previously been employed to detect SNPs under positive selection in immune-related genes (interferons and Toll-like receptors) within European and Asian human populations (Barreiro et al. 2009; Manry et al. 2011). The positive selection of the derived allele *TLR2* SNP 376A (126Thr) in European pig population based on within population tests is likely due to selective pressure mediated by bacterial infectious agents encountered by the European wild boars following divergence from their Asian counterparts. Furthermore, the conservation of the ancestral allele across the three wild pig relatives, none of which have origins in the European continent, suggests that the derived allele has arisen in response to selective pressure pertaining to Europe. In humans, clinical genetic studies have indicated a role of *TLR2* coding region polymorphisms in immune response to bacteria (Kang and Chae 2001; Schröder and Schumann 2005), demonstrating the action of selective pressure of bacterial origin on *TLR2* from a clinical perspective.

The estimated age of the *TLR2* 126Thr variant, its presence in European wild boars, European domestic pigs, Asian wild boars, and Asian domestic in this study, supports a situation where the selective pressure may have been of an ancient nature and present prior to the domestication process. The variant is likely to be of adaptive value to both European wild boars and their domesticated counterparts as they share some common bacterial agents (Meng et al. 2009), some of which may have persisted over extended periods. In this regard, one can expect that the selective pressure experienced by the European wild boars will persist in the domestic pigs. This may partly explain the high frequency of the derived allele in both European wild boars and domestic pigs. Widespread and long lasting gene have been reported between wild boars and domestic pigs across Eurasia (Chen et al. 2013; Frantz et al. 2015) and this can also explain the high frequency of the derived allele in both European wild boars and domestic pigs. Consistent with our observation of high frequency of a derived allele in both European wild boars and domestic pigs probably in response to pathogen-mediated selective pressure, genetic variability in wild boar populations has been detected to be preserved in local domestic breeds at genomic sites with potential phenotypic effects (Herrero-Medrano et al. 2014). The European domestic pig breeds involved in this study included both local and commercial breeds that have experienced artificial selective pressures of different intensities. For example, Charto Murciano, Cinta Senese, Cassertana, and Mangalitsa are local breeds and are not subjected to intense artificial selective pressures as experienced by commercial pigs like Pietrain, Duroc, and Landrace (Herrero-Medrano et al. 2014). These differences have however not resulted in much variation at the locus under positive selection as apart from one Landrace breed that was heterozygous, all other European domestic pigs were homozygous for the derived allele. Therefore, as mentioned earlier, the selective pressure responsible for the positive selective might have been in place before the domestication process. The estimated age of 163,000 ± 80,000 years of the derived allele under positive selection coincides with the late middle Pleistocene periods when there was an initiation of European wild boar expansion (about 190,000 years ago (Fang and Andersson 2006)) and when *S. scrofa* was spreading from southeastern to northeastern area of the Asian continent (about 140,000 to 253,000 years ago (Watanobe et al. 2003)). This would have provided the necessary environment for the spread of infectious diseases that would lead to adaptation at host genes. Our analysis revealed high F_ST_ values for certain *TLR* alleles between European and Asian pig populations with the highest genetic differentiation detected for *TLR2* SNP G376A (Ala126Thr). This may be due to different selective pressures associated with each population given that historically different continental populations have been exposed to different infectious agents (Troy et al. 2001; Novembre and Di Rienzo 2009). *TLR2* mediates host immune response to gram-positive bacteria, and in the case of pigs, gram positive bacteria challenges peculiar to specific continents have been documented. For example, the methicillin-resistant *Staphylococcus aureus* sequence type (ST) 398 have been detected to be highly prevalent among pigs in Europe and North America whereas ST9 is predominant in Asia (Hasman et al. 2010; Asai et al. 2012; Jamrozy et al. 2012). Such heterogeneous selective pressures across populations can result in positive selection for resistance alleles in certain populations. A similar approach of genetic differentiation (F_ST_) between populations has been used to detect geographically restricted adaptation at type III interferons in European and Asian human populations (Manry et al. 2011). In a comparison of European and Chinese pig populations utilizing F_ST_ outlier tests, the *TLR4* g.7485C have been shown to be under positive selection (Chen et al. 2013). Two haplotypes with highest frequencies are differentiated at *TLR2* SNP 376 (Ala126 for major Asian haplotype and 126Thr for the major European haplotype), further supporting a possible role of positive selection at this site.

Even though the Swiss Prot database did not indicate that *TLR2* site 126 is within a functionally relevant region of the *TLR2* protein, the LRR4 within which *TLR2* site 126 is located may be of functional relevance given that it contains *TLR2* site 136, where amino acid substitution (Pro136Ala) is associated with the prevalence of pneumonia in pigs (Uenishi et al. 2011). Therefore, one can speculate that *TLR2* Ala126Thr can be of medical relevance to porcine diseases. Non-synonymous SNPs in LRR domains have been suggested to dramatically alter the ability of the molecule to identify extracellular pathogens (Fujita et al. 2003). The nonsynonymous nature of the *TLR2* SNP G376A substitution, which causes a change of amino acid property from a non-polar to a polar amino acid (Ala126Thr) suggests that the substitution may be important in adaptation of European pigs. The location of *TLR2* 126Thr within the alpha helices of the 3D structure of *TLR1/TLR2* complex and at the N-terminal domain of the *TLR2* protein suggests it is important for ligand detection for a variety of ligands including lipoteichoic acid and peptidoglycan (Mitsuzawa et al. 2001; Meng et al. 2003). The “deleterious” nature of the threonine to alanine substitution at *TLR2* site 126 as predicted by SIFT further suggests this site is functionally relevant. A previous study investigating the effect of known polymorphisms in porcine TLRs on the recognition of *Salmonella enterica* serovar Choleraesusis (SC) did not implicate TLR2 SNP G376A in attenuating responses to SC (Shinkai et al. 2011). However, recognition of other pathogens known to affect pigs may be affected by TLR2 SNP G376A and thus requires further investigation.

Results presented here suggests a role of pathogen-mediated selective pressures among pig populations in driving the differentiation at *TLR2* SNP G376A (Ala126Thr). Future experimental functional analyses are required to determine how such SNP variant affect porcine immune response. A recent study (Tschirren et al. 2013) involving a wild rodent population has identified an association between *Borrelia* infection and haplotypes carrying the variants Ala and Thr (*TLR2* Thr276Ala) located within the ectodomain (Tschirren et al. 2013). The study of Tschirren et al. (2013) thus has demonstrated a role of alanine-threonine substitutions within *TLR2* in infectious diseases.

## Conclusions

In conclusion, this study provides evidence, based on within and between population tests, that European wild boars and domestic pigs show evidence of adaptation which is reflected in *TLR2* as signatures of selection, whereas no such evidence was observed in Asian wild boars and domestic breeds. Thus, our study suggests that *TLR2* 126Thr present in European wild boars, European domestic pigs, Asian wild boars, and Asian domestic pigs has evolved under positive selection within the European pigs involved in this study, probably in response to pathogen-mediated selective pressures. Experimental studies designed to investigate the role of the *TLR2* 126Thr in ligand binding and subsequent immune response are needed.

## Electronic supplementary material

Table S1 Summary of TLR ectodomain studied (DOCX 13 kb)

Table S2 Genomic coordinates of noncoding genomic regions (DOCX 14 kb)

Table S3 Polymorphic positions in *TLR1*, *TLR2*, *TLR3*, *TLR6*, *TLR7*, and *TLR8* in wild boars and domestic pigs (DOCX 19 kb)

Table S4 Haplotypes of *TLR2* ectodomain region (DOCX 13 kb)

Fig. S1 Geographic locations from where animal samples were obtained. (PDF 144 kb)

Fig. S2 Alignment of porcine *TLR2* amino acid sequences and human *TLR2* amino acid sequences to delineate LRRs, and functional domains of porcine TLRs. / ligand binding residues, d residues involved in dimerization, + residues involved in both ligand binding and dimerization. Asterisks, colons, and periods under the aligned sequences indicate complete match, strong conservation, and weaker conservation of amino acids respectively. (DOCX 15 kb)

Fig. S3 Neighbor-joining phylogeny of the partial D-loop region sequences of the mitochondria DNA. Red branches represent pigs of European origin and green branches represent pigs of Asian origin. (DOCX 31 kb)
