# Supplementary material for: Evidence for adaptation of porcine Toll-like receptors
Source: Immunogenetics. 2015 Dec 23;68:179–89. doi: 10.1007/s00251-015-0892-8 (PMC4759233; doi:10.1007/s00251-015-0892-8)
Supplement: Supplementary file 1 — Summary of TLR ectodomain studied (DOCX 13 kb) [file 251_2015_892_MOESM1_ESM.docx]

Title: Evidence for adaptation of porcine Toll-like receptors

Journal name: Immunogenetics

Author names: Kwame A. Darfour-Oduro^1^, Hendrik-Jan Megens^2^, Alfred Roca^1^, Martien A. M. Groenen^2^ and Lawrence B. Schook^1^

^1­^Department of Animal Sciences, University of Illinois, Urbana-Champaign, Illinois 61801, USA

^2^Animal Breeding and Genomics Centre, Wageningen University, Droevendaalsesteeg 1, Wageningen 6708 PB, The Netherlands

**Corresponding author:** **Lawrence B. Schook**

e-mail: [schook@illinois.edu](mailto:schook@illinois.edu)

**Table S1** Summary of TLR sequences studied

| **Gene** | **Exon no^a^** | **Genomic coordinates of sequences encoding ectodomain** | **Aligned length (bp) of sequences** |
| --- | --- | --- | --- |
| *TLR1* | 3 | 8:31628613-31630280:-1 | 1668 |
| *TLR2* | 2 | 8:79825324-79827018:-1 | 1695 |
| *TLR3* | 4 | 15:53849144-53849505:-1 | 366 |
| *TLR3* | 5 | 15:53848182-53848373:-1 | 192 |
| *TLR3* | 6 | 15:53841845-53843311:-1 | 1467 |
| *TLR6* | 2 | 8:31642930-31644612:-1 | 1683 |
| *TLR7* | 2 | X:10472926-10475370:1 | 2445 |
| *TLR8* | 4 | X:10509874-10512249:1 | 2376 |
| D-loop region of mtDNA |  |  | 598 |

**^a^**Exon encoding ectodomain
