# Supplementary material for: Evidence for adaptation of porcine Toll-like receptors
Source: Immunogenetics. 2015 Dec 23;68:179–89. doi: 10.1007/s00251-015-0892-8 (PMC4759233; doi:10.1007/s00251-015-0892-8)
Supplement: Supplementary file 2 — Genomic coordinates of noncoding genomic regions (DOCX 14 kb) [file 251_2015_892_MOESM2_ESM.docx]

Title: Evidence for adaptation of porcine Toll-like receptors

Journal name: Immunogenetics

Author names: Kwame A. Darfour-Oduro^1^, Hendrik-Jan Megens^2^, Alfred Roca^1^, Martien A. M. Groenen^2^ and Lawrence B. Schook^1^

^1­^Department of Animal Sciences, University of Illinois, Urbana-Champaign, Illinois 61801, USA

^2^Animal Breeding and Genomics Centre, Wageningen University, Droevendaalsesteeg 1, Wageningen 6708 PB, The Netherlands

**Corresponding author:** **Lawrence B. Schook**

e-mail: [schook@illinois.edu](mailto:schook@illinois.edu)

**Table S2** Genomic coordinates of noncoding genomic regions

| Chromosome number | Genomic coordinates |
| --- | --- |
| Chr1 | 1: 196113933-196115932 |
| Chr2 | 2: 24343289-24345288 |
| Chr3 | 3: 22621121-22623120 |
| Chr4 | 4: 25952645-25954644 |
| Chr5 | 5: 56514331-56516330 |
| Chr6 | 6: 106599095-106601094 |
| Chr7 | 7: 75443317-75445316 |
| Chr8 | 8: 101852934--101854933 |
| Chr9 | 9: 98181590-98183589 |
| Chr10 | 10: 41491552-41493551 |
| Chr11 | 11: 32898992-32900991 |
| Chr12 | 12: 30205604-30207603 |
| Chr13 | 13: 100433088-100435087 |
| Chr14 | 14: 45367685-45369684 |
| Chr15 | 15: 10096184-10098183 |
| Chr16 | 16: 31646612-31648611 |
| Chr17 | 17: 7579504-7581503 |
| Chr18 | 18: 38554557-38556556 |

*****The genomic coordinates are on the positive strand.
