# Supplementary material for: Evidence for adaptation of porcine Toll-like receptors
Source: Immunogenetics. 2015 Dec 23;68:179–89. doi: 10.1007/s00251-015-0892-8 (PMC4759233; doi:10.1007/s00251-015-0892-8)
Supplement: Supplementary file 3 — Polymorphic positions in TLR1, TLR2, TLR3, TLR6, TLR7, and TLR8 in wild boars and domestic pigs (DOCX 19 kb) [file 251_2015_892_MOESM3_ESM.docx]

Title: Evidence for adaptation of porcine Toll-like receptors

Journal name: Immunogenetics

Author names: Kwame A. Darfour-Oduro^1^, Hendrik-Jan Megens^2^, Alfred Roca^1^, Martien A. M. Groenen^2^ and Lawrence B. Schook^1^

^1­^Department of Animal Sciences, University of Illinois, Urbana-Champaign, Illinois 61801, USA

^2^Animal Breeding and Genomics Centre, Wageningen University, Droevendaalsesteeg 1, Wageningen 6708 PB, The Netherlands

**Corresponding author:** **Lawrence B. Schook**

e-mail: [schook@illinois.edu](mailto:schook@illinois.edu)

| **Table S3** Polymorphic positions in TLR1, TLR2, TLR3, TLR6, TLR7 and TLR8 in wild boars and domestic pigs | | | | | |
| --- | --- | --- | --- | --- | --- |
| Gene | SNP name | Allele frequency^a^ | | | |
|  |  | Wild boars | | Domestic pigs | |
|  |  | EWB (N=15) | AWB (N=5) | ED (N=25) | AD (N=22) |
| *TLR1* | A156G | 100.00 | 60.00 | 88.00 | 81.82 |
|  | C204T* | 100.00 | 90.00 | 100.00 | 90.91 |
|  | A329T* | 100.00 | 100.00 | 98.00 | 100.00 |
|  | C338T | 100.00 | 100.00 | 100.00 | 68.18 |
|  | T350C | 100.00 | 50.00 | 90.00 | 75.00 |
|  | G391A | 100.00 | 90.00 | 100.00 | 97.73 |
|  | A403G | 100.00 | 100.00 | 100.00 | 77.27 |
|  | C489T | 73.33 | 100.00 | 94.00 | 100.00 |
|  | G505A | 100.00 | 90.00 | 100.00 | 97.73 |
|  | T519C* | 100.00 | 90.00 | 100.00 | 97.73 |
|  | T532G | 100.00 | 90.00 | 100.00 | 97.73 |
|  | C537T* | 100.00 | 100.00 | 100.00 | 95.46 |
|  | C648T | 70.00 | 90.00 | 94.00 | 61.36 |
|  | G649A | 100.00 | 90.00 | 100.00 | 97.73 |
|  | G697A* | 100.00 | 100.00 | 100.00 | 95.46 |
|  | T798C | 30.00 | 100.00 | 46.00 | 97.73 |
|  | C840T | 100.00 | 60.00 | 88.00 | 59.09 |
|  | G855A | 100.00 | 60.00 | 88.00 | 61.36 |
|  | C900T* | 100.00 | 100.00 | 100.00 | 61.36 |
|  | C966T | 100.00 | 50.00 | 88.00 | 52.27 |
|  | A1095G* | 100.00 | 100.00 | 100.00 | 95.46 |
|  | C1278C | 100.00 | 50.00 | 88.00 | 56.82 |
|  | T1305C* | 100.00 | 100.00 | 100.00 | 95.46 |
|  | C1319T | 100.00 | 100.00 | 100.00 | 70.45 |
|  | A1351G | 100.00 | 70.00 | 88.00 | 61.36 |
|  | A1373G | 100.00 | 60.00 | 98.00 | 81.82 |
|  | T1499C | 100.00 | 80.00 | 86.00 | 97.73 |
|  | A1579G | 100.00 | 100.00 | 94.00 | 95.46 |
|  | G1636C* | 100.00 | 80.00 | 100.00 | 100.00 |
|  | G1641A* | 100.00 | 80.00 | 96.00 | 93.18 |
|  | C1647T* | 100.00 | 100.00 | 100.00 | 72.73 |
|  | A1657G | 100.00 | 100.00 | 96.00 | 61.36 |
|  | T1669A | 100.00 | 100.00 | 96.00 | 95.46 |
|  | G1675A | 100.00 | 90.00 | 100.00 | 38.64 |
|  | C1695T | 100.00 | 60.00 | 90.00 | 70.45 |
| *TLR2* | A159G | 100.00 | 100.00 | 100.00 | 97.73 |
|  | C198A* | 100.00 | 80.00 | 100.00 | 100.00 |
|  | C375T | 100.00 | 80.00 | 100.00 | 100.00 |
|  | A376G | 83.33 | 40.00 | 98.00 | 11.36 |
|  | C406G | 100.00 | 70.00 | 100.00 | 97.73 |
|  | C411T | 86.67 | 100.00 | 98.00 | 100.00 |
|  | C570T | 100.00 | 100.00 | 100.00 | 97.73 |
|  | T609C | 80.00 | 100.00 | 96.00 | 100.00 |
|  | G629C | 86.67 | 100.00 | 96.00 | 100.00 |
|  | A646G | 100.00 | 70.00 | 100.00 | 95.46 |
| Table S3 (Cont.)  Gene | SNP name | Allele frequency^a^ | | | |
|  |  | Wild boars | | Domestic pigs | |
|  |  | EWB (N=15) | AWB (N=5) | ED (N=25) | AD (N=22) |
| *TLR2* | A715C | 93.33 | 100.00 | 98.00 | 100.00 |
|  | A716G | 93.33 | 80.00 | 98.00 | 97.73 |
|  | G819C | 100.00 | 90.00 | 100.00 | 84.09 |
|  | G1012A | 100.00 | 90.00 | 100.00 | 93.18 |
|  | A1170G* | 96.67 | 100.00 | 100.00 | 100.00 |
|  | A1294G | 100.00 | 80.00 | 100.00 | 97.73 |
|  | C1475T | 100.00 | 100.00 | 100.00 | 97.73 |
|  | T1494C | 100.00 | 90.00 | 100.00 | 97.73 |
|  | G1510C | 100.00 | 100.00 | 100.00 | 84.09 |
|  | A1549C | 100.00 | 90.00 | 100.00 | 100.00 |
|  | G1739T* | 96.67 | 100.00 | 100.00 | 100.00 |
|  | G1746A | 100.00 | 100.00 | 100.00 | 97.73 |
| *TLR3* | G95A | 100.00 | 100.00 | 100.00 | 97.73 |
|  | G153A* | 100.00 | 80.00 | 100.00 | 100.00 |
|  | T159C | 100.00 | 80.00 | 100.00 | 100.00 |
|  | A405T | 90.00 | 100.00 | 100.00 | 100.00 |
|  | C798A* | 97.73 | 100.00 | 100.00 | 100.00 |
|  | C800T | 97.73 | 100.00 | 100.00 | 100.00 |
|  | A1116T | 100.00 | 70.00 | 82.00 | 61.36 |
|  | T1479C* | 100.00 | 100.00 | 98.00 | 100.00 |
|  | C1647T | 100.00 | 30.00 | 82.00 | 29.55 |
|  | C1722A* | 100.00 | 100.00 | 98.00 | 100.00 |
|  | G1857A* | 100.00 | 100.00 | 98.00 | 100.00 |
|  | G1872A | 100.00 | 80.00 | 84.00 | 47.73 |
| *TLR6* | C133T | 100.00 | 80.00 | 100.00 | 97.73 |
|  | G228A | 100.00 | 80.00 | 76.00 | 93.18 |
|  | A266G | 100.00 | 30.00 | 66.00 | 11.36 |
|  | C341T | 100.00 | 100.00 | 100.00 | 95.46 |
|  | G663T | 56.67 | 100.00 | 92.00 | 100.00 |
|  | A882G* | 100.00 | 80.00 | 98.00 | 95.46 |
|  | G919C* | 100.00 | 80.00 | 100.00 | 100.00 |
|  | C931T* | 100.00 | 100.00 | 98.00 | 100.00 |
|  | G977A | 100.00 | 100.00 | 98.00 | 100.00 |
|  | C1061T | 100.00 | 90.00 | 76.00 | 90.91 |
|  | C1124G | 96.67 | 100.00 | 100.00 | 100.00 |
|  | A1259G | 100.00 | 70.00 | 88.00 | 15.91 |
|  | G1284A | 100.00 | 80.00 | 80.00 | 93.18 |
|  | T1329C* | 100.00 | 80.00 | 100.00 | 100.00 |
|  | A1354G | 100.00 | 80.00 | 82.00 | 97.73 |
|  | G1376A | 100.00 | 20.00 | 78.00 | 77.27 |
|  | G1391A | 100.00 | 100.00 | 98.00 | 100.00 |
|  | G1438C | 100.00 | 90.00 | 86.00 | 95.46 |
|  | C1502T | 86.67 | 0.00 | 74.00 | 11.36 |
|  | A1660G | 100.00 | 80.00 | 98.00 | 95.46 |
|  | G1678A | 100.00 | 80.00 | 88.00 | 29.55 |
|  | T1698C | 93.33 | 60.00 | 80.00 | 34.09 |
|  | G1729A* | 100.00 | 80.00 | 100.00 | 100.00 |
| Table S3 (Cont.)  Gene | SNP name | Allele frequency^a^ | | | |
|  |  | Wild boars | | Domestic pigs | |
|  |  | EWB (N=15) | AWB (N=5) | ED (N=25) | AD (N=22) |
| *TLR7* | G129A* | 100.00 | 100.00 | 100.00 | 94.12 |
|  | A357G | 100.00 | 85.71 | 100.00 | 73.53 |
|  | C465T* | 100.00 | 100.00 | 100.00 | 97.06 |
|  | C520T | 100.00 | 71.43 | 100.00 | 100.00 |
|  | T663G* | 100.00 | 100.00 | 97.56 | 100.00 |
|  | T792C* | 100.00 | 85.71 | 100.00 | 97.06 |
|  | C936T* | 100.00 | 100.00 | 100.00 | 97.06 |
|  | C1019A | 78.26 | 71.43 | 65.85 | 100.00 |
|  | A1031G* | 100.00 | 100.00 | 100.00 | 94.12 |
|  | A1129C* | 100.00 | 100.00 | 100.00 | 97.06 |
|  | G1319A | 100.00 | 100.00 | 100.00 | 82.35 |
|  | T1413C | 91.30 | 57.14 | 100.00 | 52.94 |
|  | C1479A* | 100.00 | 85.71 | 100.00 | 100.00 |
|  | C1633T | 100.00 | 100.00 | 100.00 | 88.24 |
|  | C1914T* | 100.00 | 100.00 | 100.00 | 82.35 |
|  | T1917C* | 100.00 | 100.00 | 100.00 | 97.06 |
|  | G2034A | 100.00 | 85.71 | 97.56 | 76.47 |
|  | C2160T | 100.00 | 85.71 | 97.56 | 79.41 |
|  | A2232G | 78.26 | 100.00 | 63.41 | 100.00 |
| *TLR8* | C99T | 65.22 | 100.00 | 31.71 | 100.00 |
|  | G124A | 100.00 | 100.00 | 100.00 | 79.41 |
|  | G156T | 100.00 | 85.71 | 100.00 | 88.24 |
|  | C177A | 100.00 | 100.00 | 100.00 | 79.41 |
|  | T199C | 34.78 | 57.14 | 73.17 | 100.00 |
|  | A273C* | 100.00 | 100.00 | 97.56 | 100.00 |
|  | C309T* | 100.00 | 100.00 | 100.00 | 88.24 |
|  | A423G | 34.78 | 100.00 | 73.17 | 100.00 |
|  | A534C | 26.09 | 85.71 | 70.73 | 88.24 |
|  | A570T | 30.43 | 100.00 | 75.61 | 85.29 |
|  | C636T | 100.00 | 100.00 | 100.00 | 64.71 |
|  | G864T | 100.00 | 71.43 | 97.56 | 94.12 |
|  | T907C | 39.13 | 42.86 | 73.17 | 61.76 |
|  | T1150A* | 100.00 | 85.71 | 100.00 | 100.00 |
|  | C1281T* | 100.00 | 57.14 | 100.00 | 100.00 |
|  | A1593G* | 100.00 | 100.00 | 100.00 | 88.24 |
|  | T1605C | 100.00 | 71.43 | 97.56 | 88.24 |
|  | G1647A* | 100.00 | 85.71 | 100.00 | 100.00 |
|  | T1710A | 69.57 | 85.71 | 26.83 | 100.00 |
|  | C1740T | 100.00 | 85.71 | 100.00 | 52.94 |
|  | A2144G* | 95.65 | 100.00 | 100.00 | 100.00 |
|  | C2338T | 100.00 | 85.71 | 100.00 | 82.35 |
|  | A2394G | 82.61 | 71.43 | 31.71 | 88.24 |
|  | C2397T* | 100.00 | 85.71 | 100.00 | 82.35 |
|  | G2439A | 100.00 | 100.00 | 90.24 | 100.00 |

^a^The frequency of the first allele. The number in SNP name indicates nucleotide position of SNPs within TLR coding sequences. EWB European wild boar, AWB Asian wild boar, ED European domestic pig, AD Asian domestic pig

N number of animals. *SNPs that were not reported in the studies of Shinkai et al., (2006); Uenishi et al., (2009) and Bergman et al., (2010). All other SNPs have been previously reported.
