# Supplementary material for: Evidence for adaptation of porcine Toll-like receptors
Source: Immunogenetics. 2015 Dec 23;68:179–89. doi: 10.1007/s00251-015-0892-8 (PMC4759233; doi:10.1007/s00251-015-0892-8)
Supplement: Supplementary file 4 — Haplotypes of TLR2 ectodomain region (DOCX 13 kb) [file 251_2015_892_MOESM4_ESM.docx]

Title: Evidence for adaptation of porcine Toll-like receptors

Journal name: Immunogenetics

Author names: Kwame A. Darfour-Oduro^1^, Hendrik-Jan Megens^2^, Alfred Roca^1^, Martien A. M. Groenen^2^ and Lawrence B. Schook^1^

^1­^Department of Animal Sciences, University of Illinois, Urbana-Champaign, Illinois 61801, USA

^2^Animal Breeding and Genomics Centre, Wageningen University, Droevendaalsesteeg 1, Wageningen 6708 PB, The Netherlands

**Corresponding author:** **Lawrence B. Schook**

e-mail: [schook@illinois.edu](mailto:schook@illinois.edu)

**Table S4**. Haplotypes of TLR2 sequences encoding the ectodomain

| **Haplotype** | **SNPs** | | | | | | | | | |
| --- | --- | --- | --- | --- | --- | --- | --- | --- | --- | --- |
|  | 376 | 411 | 609 | 629 | 646 | 715 | 716 | 819 | 1012 | 1510 |
| H_1 (70) | A | C | T | G | A | A | A | G | G | G |
| H_2 (3) | G | T | T | C | A | C | G | G | G | G |
| H_4 (4) | G | C | T | G | A | A | A | C | G | C |
| H_5 (6) | A | C | C | G | A | A | A | G | G | G |
| H_8 (33) | G | C | T | G | A | A | A | G | G | G |
| H_10 (2) | G | C | T | G | A | A | A | C | A | C |
| H_13 (2) | A | C | T | C | A | A | A | G | G | G |
| H_14 (2) | G | C | T | G | G | A | A | G | G | G |

Numbers in brackets are frequencies of the haplotypes.
