# Supplementary material for: Evidence for adaptation of porcine Toll-like receptors
Source: Immunogenetics. 2015 Dec 23;68:179–89. doi: 10.1007/s00251-015-0892-8 (PMC4759233; doi:10.1007/s00251-015-0892-8)

Title: Evidence for adaptation of porcine Toll-like receptors

Journal name: Immunogenetics

Author names: Kwame A. Darfour-Oduro[1], Hendrik-Jan Megens[2], Alfred Roca[1], Martien A. M. Groenen[2] and Lawrence B. Schook[1]

[1]Department of Animal Sciences, University of Illinois, Urbana-Champaign, Illinois 61801, USA

[2]Animal Breeding and Genomics Centre, Wageningen University, Droevendaalsesteeg 1, Wageningen 6708 PB, The Netherlands

Corresponding author: Lawrence B. Schook
e-mail: schook@illinois.edu

Fig. S1

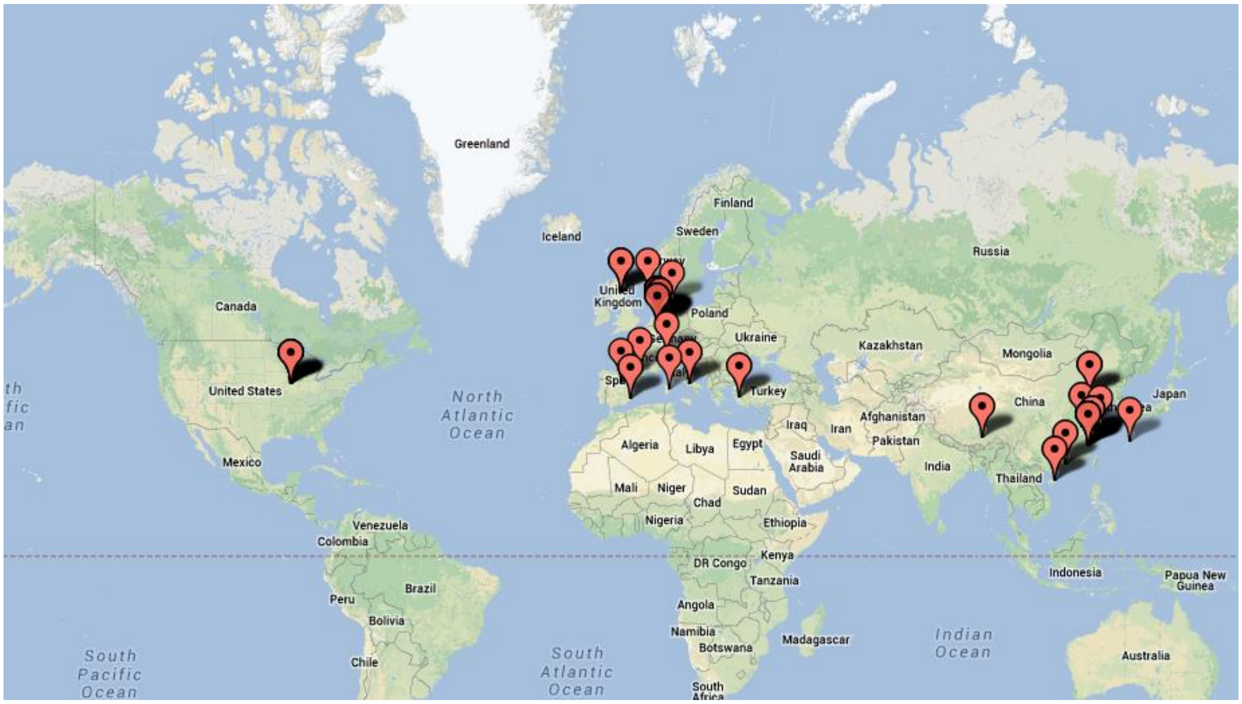

Supplement: Supplementary file 5 — Geographic locations from where animal samples were obtained. (PDF 144 kb) [file 251_2015_892_MOESM5_ESM.pdf]
