# Supplementary material for: Evidence for adaptation of porcine Toll-like receptors
Source: Immunogenetics. 2015 Dec 23;68:179–89. doi: 10.1007/s00251-015-0892-8 (PMC4759233; doi:10.1007/s00251-015-0892-8)
Supplement: Supplementary file 6 — Alignment of porcine TLR2 amino acid sequences and human TLR2 amino acid sequences to delineate LRRs, and functional domains of porcine TLRs. / ligand binding residues, d residues involved in dimerization, + residues involved in both ligand binding and dimerization. Asterisks, colons, and periods under the aligned sequences indicate complete match, strong conservation, and weaker conservation of amino acids respectively. (DOCX 15 kb) [file 251_2015_892_MOESM6_ESM.docx]

Title: Evidence for adaptation of porcine Toll-like receptors

Journal name: Immunogenetics

Author names: Kwame A. Darfour-Oduro^1^, Hendrik-Jan Megens^2^, Alfred Roca^1^, Martien A. M. Groenen^2^ and Lawrence B. Schook^1^

^1­^Department of Animal Sciences, University of Illinois, Urbana-Champaign, Illinois 61801, USA

^2^Animal Breeding and Genomics Centre, Wageningen University, Droevendaalsesteeg 1, Wageningen 6708 PB, The Netherlands

**Corresponding author:** **Lawrence B. Schook**

e-mail: [schook@illinois.edu](mailto:schook@illinois.edu)

**Fig. S2**

LRRNT LRR1

Human_TLR2 MPHTLWMVWVLGVIISLSKEESSNQAS-LSCDRNGICKGSSGSLNSIPSGLTEAVKSLDL 59

Porcine_TLR2 MPCALWTAWVLGIVISLSKEGAPHQASSLSCDPAGVCDGRSRSLSSIPSGLTAAVKSLDL 60

** :** .****::****** :.:*** **** *:*.* * **.******* *******

LRR2 LRR3

Human_TLR2 SNNRITYISNSDLQRCVNLQALVLTSNGINTIEEDSFSSLGSLEHLDLSYNYLSNLSSSW 119

Porcine_TLR2 SNNRIAYVGSSDLRKCVNLRALRLGANSIHTVEEDSFSSLGSLEHLDLSYNHLSNLSSSW 120

*****:*:..***::****:** * :*.*:*:*******************:********

LRR4 LRR5 LRR6

Human_TLR2 FKPLSSLTFLNLLGNPYKTLGETSLFSHLTKLQILRVGNMDTFTKIQRKDFAGLTFLEEL 179

Porcine_TLR2 FKSLSTLKFLNLLGNPYKTLGEAPLFSHLPNLRILKIGNNDTFPEIQAKDFQGLTFLQEL 180

**.**:*.**************:.*****.:*:**::** ***.:** *** *****:**

LRR7 LRR8

Human_TLR2 EIDASDLQSYEPKSLKSIQNVSHLILHMKQHILLLEIFVDVTSSVECLELRDTDLDTFHF 239

Porcine_TLR2 EIGASHLQRYAPKSLRSIQNISHLILHMRRPALLPKIFVDLLSSLEYLELRNTDFSTFNF 240

**.**.** * ****:****:*******:: ** :****: **:* ****:**:.**:*

LRR9 / / LRR10 / / / //

Human_TLR2 SELSTGETNSLIKKFTFRNVKITDESLFQVMKLLNQISGLLELEFDDCTLNGVGNFRASD 299

Porcine_TLR2 SDVSINEHCTVMKKFTFRKAEITDASFTEIVKLLNYVSGALEVEFDDCTLNGRGDLSTSA 300

*::* .* :::******:.:*** *: :::**** :** **:********* *:: :*

LRR11 LRR12

/ / / /d/ ddd+// / / / /d d/++/+ /

Human_TLR2 NDRVIDPGKVETLTIRRLHIPRFYLFYDLSTLYSLTERVKRITVENSKVFLVPCLLSQHL 359

Porcine_TLR2 LDTIKSLGNVETLTVRRLHIPQFFLFYDLRSIYSLTGAVKRITIENSKVFLVPCSLSQHL 360

* : . *:*****:******:*:***** ::**** *****:********** *****

LRR13 d d dddd d LRR14 d LRR15

Human_TLR2 KSLEYLDLSENLMVEEYLKNSACEDAWPSLQTLILRQNHLASLEKTGETLLTLKNLTNID 419

Porcine_TLR2 KSLEYLDLSENLMSEEYLKNSACEHAWPFLHTLILRQNHLKSLEKTGEVLVTLKNLTNLD 420

************* **********.*** *:********* *******.*:*******:*

LRR16 LRR17 LRR18

Human_TLR2 ISKNSFHSMPETCQWPEKMKYLNLSSTRIHSVTGCIPKTLEILDVSNNNLNLFSLNLPQL 479

Porcine_TLR2 ISKNNFDSMPETCQWPEKMKYLNLSSTRIHSLTHCLPQTLEVLDISNNNLNSFSLSLPQL 480

****.*.************************:* *:*:***:**:****** ***.****

LRR19 LRR20

Human_TLR2 KELYISRNKLMTLPDASLLPMLLVLKISRNAITTFSKEQLDSFHTLKTLEAGGNNFICSC 539

Porcine_TLR2 KELYISRNKLKTLPDASFLPMLSVLRISRNTINTFSKEQLDSFQKLKTLEAGGNNFICSC 540

********** ******:**** **:****:*.**********:.***************

LRRCT

Human_TLR2 EFLSFTQEQQALAKVLIDWPANYLCDSPSHVRGQQVQDVRLSVSECHRTALVSGMCCALF 599

Porcine_TLR2 DFLSFTQGQQALAQVLSDWPENYLCDSPSHVRGQRVQDTRLSLTECHRVAVVSVVCCALF 600

:****** *****:** *** *************:***.***::****.*:** :*****

Human_TLR2 LLILLTGVLCHRFHGLWYMKMMWAWLQAKRKPRKAPSRNICYDAFVSYSERDAYWVENLM 659

Porcine_TLR2 LLLLLTGALCHHFHGLWCMKMMWAWLQAKRKPRKAPRRDVCYDAFVSYSEQDSYWVENLM 660

**:****.***:***** ****************** *::**********:*:*******

Human_TLR2 VQELENFNPPFKLCLHKRDFIPGKWIIDNIIDSIEKSHKTVFVLSENFVKSEWCKYELDF 719

Porcine_TLR2 VQELEHFQPPFKLCLHKRDFIPGKWIIDNIIDSIEKSQKTIFVLSENFVKSEWCKYELDF 720

*****:*:*****************************:**:*******************

Human_TLR2 SHFRLFDENNDAAILILLEPIEKKAIPQRFCKLRKIMNTKTYLEWPMDEAQREGFWVNLR 779

Porcine_TLR2 SHFRLFDENDDTAILILLEPIEKKTIPQRFCKLRKIMNTRTYLEWPADETQREGFWLNLR 780

*********:*:************:**************:****** **:******:***

Human_TLR2 AAIKS 784

Porcine_TLR2 AAIKS 785
