# Supplementary material for: Evidence for adaptation of porcine Toll-like receptors
Source: Immunogenetics. 2015 Dec 23;68:179–89. doi: 10.1007/s00251-015-0892-8 (PMC4759233; doi:10.1007/s00251-015-0892-8)

Title: Evidence for adaptation of porcine Toll-like receptors

Journal name: Immunogenetics

Author names: Kwame A. Darfour-Oduro^1^, Hendrik-Jan Megens^2^, Alfred Roca^1^, Martien A. M. Groenen^2^ and Lawrence B. Schook^1^

^1­^Department of Animal Sciences, University of Illinois, Urbana-Champaign, Illinois 61801, USA

^2^Animal Breeding and Genomics Centre, Wageningen University, Droevendaalsesteeg 1, Wageningen 6708 PB, The Netherlands

**Corresponding author:** **Lawrence B. Schook**

e-mail: [schook@illinois.edu](mailto:schook@illinois.edu)

Fig. S3


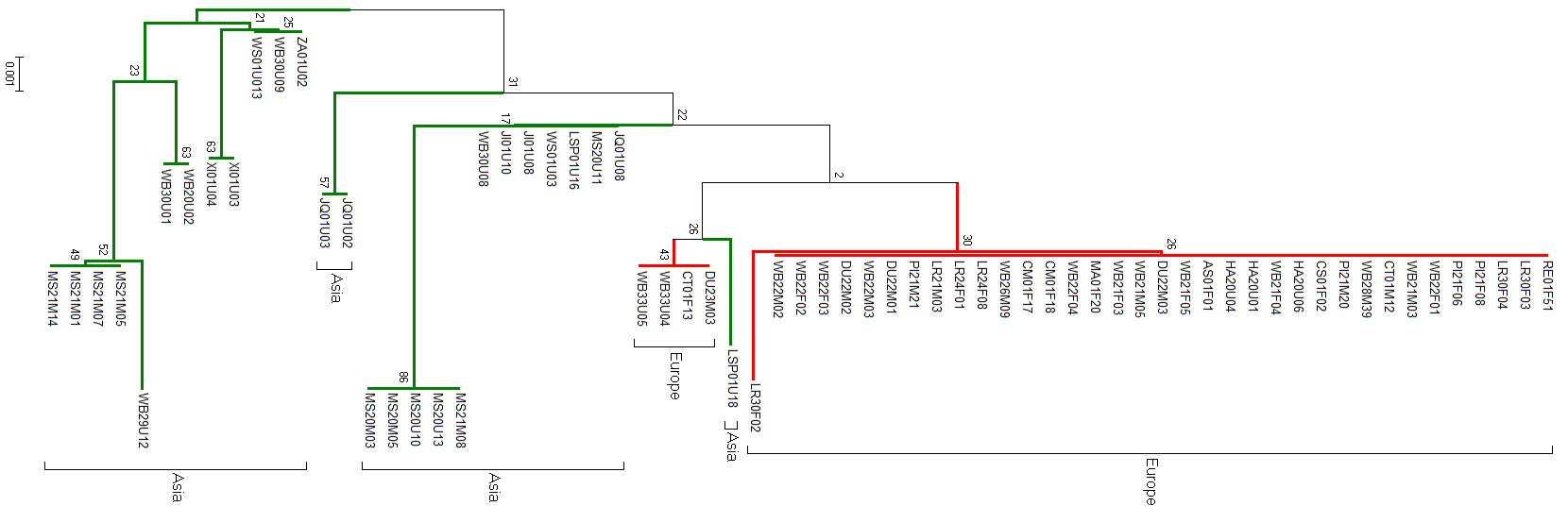

Supplement: Supplementary file 7 — Neighbor-joining phylogeny of the partial D-loop region sequences of the mitochondria DNA. Red branches represent pigs of European origin and green branches represent pigs of Asian origin. (DOCX 31 kb) [file 251_2015_892_MOESM7_ESM.docx]
